# Chronic Deltoid Insufficiency in Stage IV Adult Acquired Flatfoot Deformity: Do We Have a Good Answer?

**DOI:** 10.7759/cureus.62711

**Published:** 2024-06-19

**Authors:** Jacob C Tiell, Matias Malkamaki, Patrick O'Connor, Nicholas C Cheney

**Affiliations:** 1 Orthopedic Surgery, Ohio University Heritage College of Osteopathic Medicine, Dublin, USA; 2 Biomedical Sciences, Ohio University Heritage College of Osteopathic Medicine, Athens, USA; 3 Orthopedic Surgery, OrthoNeuro, Columbus, USA

**Keywords:** foot and ankle, orthopedic surgery, aafd, deltoid ligament, flatfoot

## Abstract

Stage IV adult acquired flatfoot deformity (AAFD) with secondary chronic deltoid ligament insufficiency is a challenging deformity to treat, with minimal consensus in the literature concerning its surgical management. Many surgical treatment options have been described, including joint-sparing techniques, fusions, osteotomies, and even arthroplasties. However, questions remain as to what, if any, treatment is optimal. This contribution reviews studies on surgical treatments for stage IV AAFD with deltoid ligament failure and provides a critical analysis regarding the quality of outcomes reported for those different treatment options.

PubMed and Google Scholar databases were searched between June 1, 2022, and August 15, 2022, for studies published between 1990 and 2022 that describe the treatment of stage IV AAFD with deltoid ligament insufficiency. Articles included in the study focused on subjects with stage IV AAFD and associated deltoid ligament insufficiency undergoing surgical correction. Exclusion criteria included stage I, II, and III AAFD, as well as deltoid ligament repair following acute injury/rupture.

Nine studies covering five different treatment options for patients with stage IV AAFD and chronic deltoid insufficiency were included, with minimal overlap in outcome measures used to assess the efficacy of the procedure. Triple arthrodesis with deltoid ligament reconstruction resulted in a 62.5% (5/8) success rate with a residual tibiotalar (TT) angulation of 2° (success defined as <3°). Tibiotalar arthrodesis of four patients resulted in an average post-operative tibiotalar angulation of 4.8° with all patients showing progressive destabilization of the hindfoot complex at 12-18 year follow-ups. Deltoid arthroscopic laminoplasty (Brostrom) resulted in an increased American Orthopaedic Foot and Ankle Society (AOFAS) score from 49.7 pre-op to 91.9 post-op. There was no long-term follow-up of these patients. Deltoid ligament reconstruction using autografts of the peroneus longus resulted in a post-operative valgus of 2.1° in one study and <5° in another. Deltoid ligament reconstruction using an anterior tibial tendon autograft resulted in a gain of 126.4 + 40.2% in stiffness compared to an intact ligament. Twinfix suture anchors resulted in a post-operative hindfoot angle averaging 5.3°. Combined deltoid and spring ligament reconstruction resulted in a 5.1° valgus angulation.

There is currently no standard of care or clinical consensus regarding surgical treatment for stage IV AAFD with deltoid insufficiency. Several studies imply that mild valgus malalignment around the tibiotalar joint can result in satisfactory outcomes. A few studies even deemed <5° of valgus tilt post-operatively successful. However, it has been described that any imbalance in tibiotalar tilt is a significant risk factor for progressive arthritis and future ligamentous failure. No treatment option was able to correct valgus tilt to an anatomical standard (i.e., to normal anatomy). These varied findings, along with the lack of consensus on post-surgical measures to assess efficacy, are worrisome and emphasize the need for better surgical options. Moreover, there is a critical need for additional research on the long-term outcomes following stage IV AAFD and deltoid insufficiency repair, particularly, as over five million people in the United States and 10% of the geriatric population are affected by AAFD with a risk of progressing to stage IV.

## Introduction and background

Adult acquired flatfoot deformity (AAFD) was first described as posterior tibial tendon dysfunction and represents a complex pathology defined by the collapse of the medial longitudinal arch of the foot with continued progressive deformity of the foot and ankle. This debilitating condition currently affects up to five million people within the United States alone [1,2] and 10% of the geriatric population [3]. The initial pathogenesis and progression of AAFD involves a cascade of degenerative changes in the distal lower extremity.

**Figure 1:**
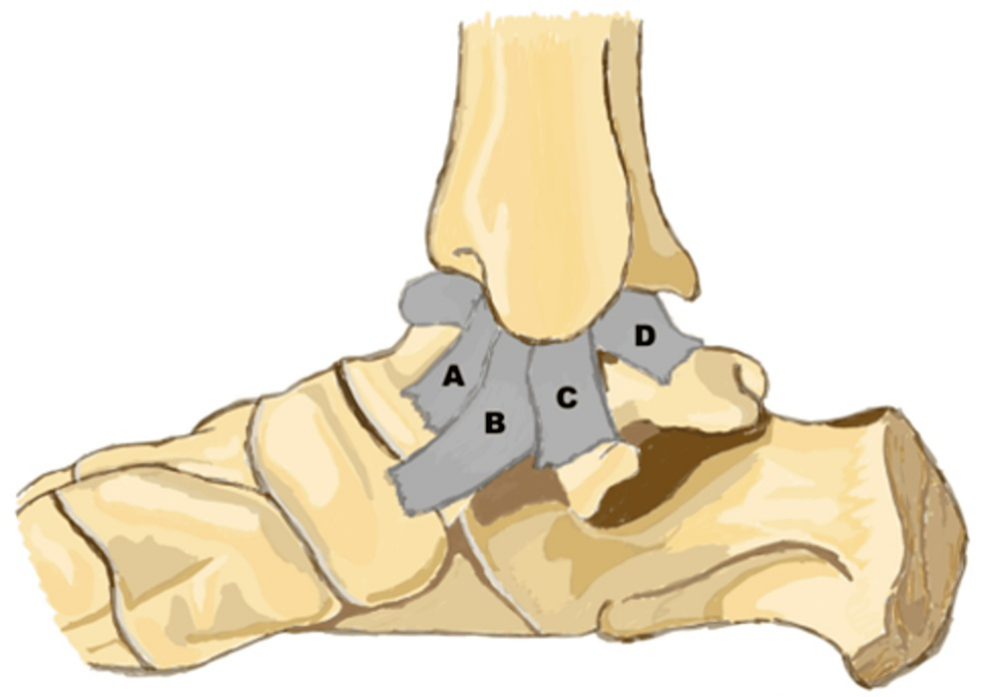
Deltoid ligament illustration Deltoid ligament illustration demonstrating the (A) superficial tibionavicular, (B) tibiospring, and (C) tibiocalcaneal ligaments. The deep layer is composed of anterior tibiotalar (not shown) and (D) posterior tibiotalar ligaments. Used with permission from the Radiological Society of North America: Flores DV, Mejía Gómez C, Fernández Hernando M, Davis MA, Pathria MN: Adult Acquired Flatfoot Deformity: Anatomy, Biomechanics, Staging, and Imaging Findings. Radiographics. 2019, 39:1437-60. 10.1148/rg.2019190046 [4] Permission conveyed through Copyright Clearance Center, Inc.

The deltoid ligament is the primary static stabilizer of the medial ankle. This structure arises from the medial malleolus and has both deep and superficial layers (Figure 1). The superficial layer is composed of the tibionavicular ligament, tibiospring ligament, and tibiocalcaneal ligament. This superficial layer is the primary support against tibiotalar (TT) valgus angulation [2]. The deep layer of the deltoid ligament is composed of the anterior and posterior tibiotalar ligaments, both of which prevent axial rotation of the talus. Any deficiency in the deltoid ligament can lead to valgus positioning of the talus within the ankle mortise either with or without axial rotation of the talus.

The Bluman-Myerson classification system (Table 1) [2,4-6] is widely accepted among clinicians for consideration of adult acquired flatfoot deformity. Stage I involves tibial tendon tenosynovitis without arch collapse. Stage II is a flexible deformity involving arch collapse and is further divided into stage IIA (valgus deformity of hindfoot but no midfoot abduction) and stage IIB (midfoot abduction). Stage III is a rigid deformity with hindfoot valgus and forefoot abduction. Stage IV is ankle valgus secondary to deltoid ligament attenuation and is subclassified into stages IVA and IVB. Stage IVA is a flexible flatfoot where the hindfoot is in valgus with flexible ankle valgus and no significant ankle arthritis. Stage IVB is a rigid foot deformity. The hindfoot is in valgus with a rigid valgus ankle and/or significant tibiotalar arthritis.

**Table 1:** Simplified Bluman-Myerson classification Simplification of the Bluman-Myerson classification system for AAFD with disease progression and corresponding treatment options. Initially described by Bluman et al. [6]. AAFD: adult acquired flatfoot deformity, PTT: posterior tibial tendon

Simplified Bluman-Myerson classification				
Stage		Deformity	Disease progression	Treatment
I		None	PTT tendinosis or tenosynovitis	Conservative initially
			Functional tendon	Tenosynovectomy
II	IIA	Flexible moderate deformity (<40% of the talar head uncovered)	Tendinosis or low-to moderate-grade tear of PTT	Orthoses
			Laxity of spring ligament	Tendon transfer
				Medializing calcaneal osteotomy
	IIB	Flexible severe deformity (>40% of the talar head uncovered or subtalar impingement)	High-grade tear of PTT	Subtalar arthrodesis
			Incomplete spring ligament	Medial column stabilizing procedure
			Sinus tarsi syndrome	Consider adding lateral column lengthening with or without spring ligament reconstruction
III		Subtalar osteoarthritis	Subtalar osteoarthritis	Subtalar arthrodesis or triple arthrodesis
		Lateral hindfoot impingement	Lateral hindfoot impingement	Consider adding medial ray procedure for plantar flexion of the first metatarsal
IV	IVA	Flexible tibiotalar valgus	Deltoid ligament abnormality	Flatfoot reconstruction + deltoid ligament reconstruction
	IVB	Rigid tibiotalar valgus	Tibiotalar osteoarthritis	Consider adding tibiotalar fusion or ankle arthroplasty

Stage IV AAFD with secondary chronic deltoid ligament insufficiency is a challenging valgus deformity to treat, with little to no consensus in the literature concerning its surgical management. With many techniques being used, including joint-sparing, fusions, osteotomies, and even arthroplasties, the question remains as to what, if any, treatment option is optimal. The purpose of this study was to provide a critical analysis regarding the quality of outcomes reported for the different treatment options and bring to light the need for advancement in disease management.

## Review

Aim, design, and setting of the study

The goal of this study was to (1) identify the surgical methods for correcting chronic deltoid insufficiency in stage IV adult acquired flatfoot deformity (AAFD) and (2) evaluate effectiveness at providing long-term improvement in various subjective and objective rating scales.

Eligibility criteria for literature review

A study needed to include an individual group of study patients who met the criteria for chronic deltoid insufficiency in stage IV AAFD. Studies needed to describe a procedure for correcting stage IV adult acquired flatfoot deformity. Studies needed to have some form of a subjective rating scale of symptom improvement and/or some form of an objective measure of ankle realignment. Studies were not eliminated based on sample size, duration of study time post-op, or number/type of rating scale(s) used.

Information sources

The literature survey was conducted between June 1, 2022, and January 14, 2024. Sources for the literature review included PubMed, Google Scholar, linkinghub.elsevier.com, ncbi.nlm.nih.gov, journals.sagepub.com, onlinelibrary.wiley.com, bmj.com, journals.lww.com, jfootankleres.biomedcentral.com, josr-online.biomedcentral.com, meridian.allenpress.com, dovepress.com, ajronline.org, hmpgloballearningnetwork.com, dx.plos.org, link.springer.com, and pubs.rsna.org.

Search strategy

The following keywords and combinations of keywords were used: adolescent, adult, age factors, ankle, ankle fractures, ankle injuries, ankle joint arthrodesis, biomechanical phenomena, bone screws, cadaver, calcaneus, deltoid, dissection, female, fibula, flatfoot, follow-up studies, foot, foot deformities, acquired, foot orthoses, foot orthoses, fracture fixation, internal fractures, bone fractures, malunited gait, 20th century history, 21st century history, humans, immobilization, joint deformities, acquired joint instability, leg length inequality, ligaments, articular ligaments, lower extremity, male, metatarsal bones, middle aged, models, anatomic muscular, diseases, orthopedic procedures, orthotic devices, orthotic insoles, osteoarthritis, osteotomy pain, patient satisfaction, pes planus, postoperative complications, predictive value of tests, pressure, prognosis, prostheses and implants, prosthesis design, radiography, articular range of motion, reconstructive surgical procedures, retrospective studies, risk assessment, rotation, severity of illness index, shoe inserts, subtalar joint, suture anchors, suture techniques, syndesmosis, talus, tarsal joints, tendons, tibia, tibial fractures, time factors, treatment outcome, and wound healing.

Data items

The purpose of this study was to survey the published literature describing surgical treatments/techniques for the repair of stage IV AAFD with chronic deltoid ligament insufficiency. For this reason, the outcomes of each study, whether favorable or not, were important to include in this work. All results were compiled regardless of endpoint, subjective versus objective data, or other outcome domains.

The articles summarized in this work used one or more of the following subjective or objective measures/scores to assess outcomes. For functional outcomes, the American Orthopaedic Foot and Ankle Society (AOFAS) Ankle-Hindfoot Score, Visual Analog Scale (VAS), Foot and Ankle Outcome Score (FAOS), SF-36v2, SF-36, Mean SF-36 Mental Component, Maryland Foot Score (MFS), Mean MFS, ankle range of motion (ROM), SF-12 scores, Foot and Ankle Ability Measure (FAAM), and Patient-Reported Outcomes Measurement Information System (PROMIS) were used. For radiographic outcomes, ankle radiographs, valgus talar tilt, hindfoot alignment/angle, angle between the long axes of the talus and first metatarsal, tibiotalar valgus angulation reduction, lateral ankle joint space, talonavicular angle, Meary's angle, angular displacement at a 2Nm level torque (cadaveric), strain at tibiocalcaneal fibers of superficial deltoid ligament complex (cadaveric), deltoid ligament strains/force (cadaveric), abduction and sagittal arch in weight-bearing radiographs, and total arc of motion were used.

Study risk of bias assessment

There was no assessment of the risk of bias included in this study. The study is meant to broadly survey the literature regarding repair options for stage IV AAFD with chronic deltoid ligament insufficiency and could have been impacted by bias.

Effect measures

As many of the studies collected used widely different subjective and objective criteria to measure outcomes, they were not compared on a statistical level. The outcomes were simply compiled and are described for the reader to evaluate.

Synthesis methods

Opinions regarding the quality or significance of various treatment methods and their results are entirely subjective and are reflections of the personal opinions of the authors of the contribution and given the data collected.

Findings

Following a review of the literature, nine studies covering five different treatment options for patients with stage IV AAFD and chronic deltoid insufficiency were included (Table 2). Treatments included triple arthrodesis and its combinations, tibiotalar (TT) arthrodesis, deltoid arthroscopic ligamentoplasty (Brostrom), deltoid ligament reconstruction, and deltoid + spring ligament reconstruction.

**Table 2:** Articles that satisfied the inclusion criteria

Study title	Author	Year	Treatment studied	DOI
Minimally Invasive Deltoid Ligament Reconstruction for Stage IV Flatfoot Deformity	Jeng et al. [7]	2011	Triple arthrodesis + deltoid ligament reconstruction	10.3113/FAI.2011.0021
Peritalar Instability After Tibiotalar Fusion for Valgus Unstable Ankle in Stage IV Adult Acquired Flatfoot Deformity: Case Series	Colin et al. [8]	2013	Tibiotalar arthrodesis	10.1177/1071100713505753
Medial and Lateral Combined Ligament Arthroscopic Repair for Multidirectional Ankle Instability	Mansur et al. [9]	2021	Deltoid arthroscopic ligamentoplasty (Brostrom)	10.1177/2473011420986150
Deltoid Ligament Reconstruction With Peroneus Longus Autograft in Flatfoot Deformity	Ellis et al. [10]	2010	Deltoid ligament reconstruction - peroneus longus autograft	10.3113/FAI.2010.0781
Reconstruction of the Chronically Failed Deltoid Ligament: A New Technique	Deland et al. [11]	2004	Deltoid ligament reconstruction - peroneus longus autograft	10.1177/107110070402501107
Deltoid Ligament Reconstruction: A Novel Technique With Biomechanical Analysis	Haddad et al. [12]	2010	Deltoid ligament reconstruction - anterior tibial tendon autograft	10.3113/FAI.2010.0639
Treatment of Chronic Deltoid Ligament Injury Using Suture Anchors	Wang et al. [13]	2014	Twinfix suture anchors	10.1111/os.12125
Deltoid-Spring Ligament Reconstruction in Adult Acquired Flatfoot Deformity With Medial Peritalar Instability	Brodell et al. [14]	2019	Deltoid + spring ligament reconstruction	10.1177/1071100719839176
Peritalar Kinematics With Combined Deltoid-Spring Ligament Reconstruction in Simulated Advanced Adult Acquired Flatfoot Deformity	MacDonald et al. [15]	2020	Deltoid + spring ligament reconstruction	10.1177/1071100720929004

Triple Arthrodesis + Deltoid Ligament Reconstruction

Jeng et al. [7] combined triple arthrodesis with hamstring tendon allograft deltoid ligament reconstruction in eight patients who underwent the procedure with comparisons using both radiographic and functional outcomes. A successful outcome was defined as maintenance of 3° or less of valgus tibiotalar angulation and greater than 2 mm of lateral joint space remaining at the final follow-up. Five of eight (62.5%) patients were judged to have a successful outcome. In those, tibiotalar valgus angulation was reduced from 6.4° ± 2.9° pre-operatively to 2.0° ± 2.0° post-operation. Lateral ankle joint space was maintained at pre-operative levels, and SF-12 functional scores were equal to age-matched normative scores.

Tibiotalar Arthrodesis

Four patients underwent isolated tibiotalar arthrodesis for the treatment of valgus instability in stage IV AAFD [8]. Pre-operatively, talar tilt averaged 12° of valgus across patients. Post-operatively, talar tilt averaged 4.8° of valgus, and calcaneal offset relative to the tibial axis was in valgus misalignment in all patients ranging from 16 to 54 mm in the Saltzman view. All radiographs were weight-bearing. At the latest follow-up, all patients had shown a progressive destabilization of the hindfoot complex, resulting in valgus and pronation deformities with flattening of the arch. Patients all indicated pain over medial and lateral malleoli, as well as pain with and reduction in motion of the ankle joint. Bohay and Anderson [16] are noted here as they also used this treatment option. However, they did not provide data on patient follow-up.

Deltoid Arthroscopic Ligamentoplasty (Brostrom)

A total of 30 ankles (29 patients) undergoing deltoid arthroscopic ligamentoplasty (Brostrom) were included in a retrospective study performed by Mansur et al. [9]. All patients were evaluated for pain and function using the Visual Analog Scale (VAS) score and the American Orthopaedic Foot and Ankle Society (AOFAS) Hindfoot Score at a mean of 14.8 months follow-up. The AOFAS score was found to increase from 49.7 pre-operatively to 92.9 post-operatively. VAS score pre-operatively was 6.83 with post-operative scores averaging 0.95. Complications were identified in 16% of patients. There were no notes related to post-operative ankle alignment or any radiographic parameters.

Deltoid Ligament Reconstruction

Peroneus longus tendon autograft was included in our studies. Ellis et al. [10] performed this treatment on five patients using FAOS, SF-36v2, VAS, weight-bearing radiographs of the ankle, ankle range of motion (ROM), and hindfoot alignment as outcome measures. Pre-operative FAOS averaged 68.3 with SF-36v2 averaging 75.7 post-operatively. Valgus talar tilt pre-operation averaged 7.7° with an average correction to 2.1° post-operation. Ankle ROM averaged 47° post-operation with hindfoot alignment averaging 4° of valgus post-operation.

Deland et al. [11] also used peroneus longus tendon autografts for the treatment of chronically failed deltoid ligaments. Four out of five (80%) procedures resulted in correction of valgus tilt to 4° or less and were considered successful. One patient retained 9° of valgus tilt post-operatively. No patients were able to achieve 0° of valgus tilt. The study also reported all five patients doing well functionally at 2+ years follow-up, with only two patients describing mild limitations with walking, yet both can walk 10 blocks before feeling limited.

Another study on deltoid ligament reconstruction involved the use of a tibialis anterior tendon autograft. Haddad et al. [12] used six fresh frozen cadaveric lower extremities to evaluate this procedure with a biomechanical analysis. A motor was used to position cadavers in dorsiflexion/plantarflexion, inversion/eversion, and internal/external rotation. Angular rotations and linear translation of the tibia in three dimensions were measured for a given torque through the range of motions noted above. This resulted in angular displacement at a 2Nm level of torque that was significantly greater in the sectioned group compared to the deltoid reconstruction group in external rotation and eversion. There was no statistical difference in the deltoid intact group compared to the reconstructed group in either external rotation or eversion. The stiffness of reconstructed specimens was 126.4 + 40.2% greater compared to the intact ligament. Nonetheless, there was no significant difference in either plantar flexion or dorsiflexion between the reconstructed group and sectioned groups.

Finally, one retrospective study evaluated 17 patients who had undergone deltoid ligament reconstruction using Twinfix suture anchors [13]. The AOFAS Ankle-Hindfoot Score and radiographic parameters were used to evaluate ankles pre- and post-operatively. Radiographic measurements included the angle between the long axes of the talus and the first metatarsal and the hindfoot angle measured in a hindfoot alignment view. Ten patients had outcomes reported as "excellent," six patients as "good," and one patient as "fair." Patients had a mean follow-up of 20.1 months. The angle between the long axis of the talus and the first metatarsal pre-operatively measured 5.4° + 1.8° and post-operatively measured 4.0° + 0.9°. The hindfoot angle measured in a hindfoot alignment view was 8.2° + 2.6° pre-operatively and 5.3°​​​​​​​ + 1.3°​​​​​​​ post-operatively. AOFAS Ankle-Hindfoot scores measured 76.8 + 7.0 pre-operation and 94.1 + 3.3 post-operation. Pain was relieved in all patients, and no patients had recurrent deltoid ligament injury.

Deltoid + Spring Ligament Reconstruction

**Figure 2:**
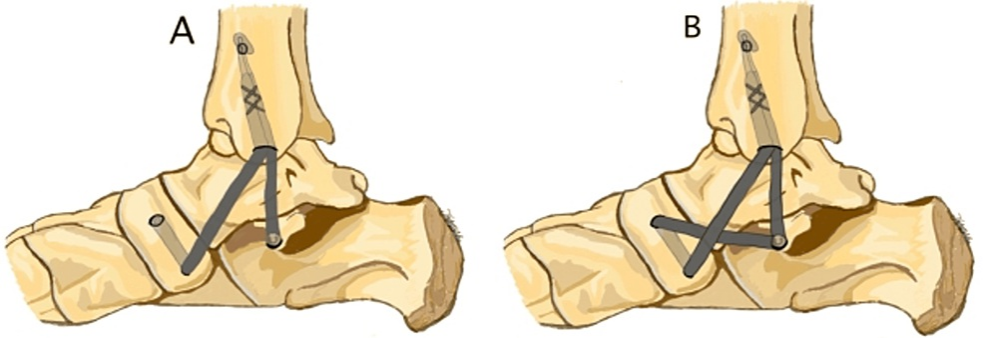
Tibiocalcaneonavicular ligament reconstruction techniques Schematic diagram of the two different tibiocalcaneonavicular ligament reconstruction techniques used by Brodell et al. [14]. (A) was used in six ankle procedures, while the technique in (B) included a figure eight passage across the navicular to the sustentaculum tali for eight ankle procedures. Reprinted with permission from SAGE Publications: Brodell JD, MacDonald A, Perkins JA, Deland JT, Oh I: Deltoid-Spring Ligament Reconstruction in Adult Acquired Flatfoot Deformity With Medial Peritalar Instability. Foot Ankle Int. 2019;40(7):753-61 [14]

**Figure 3:**
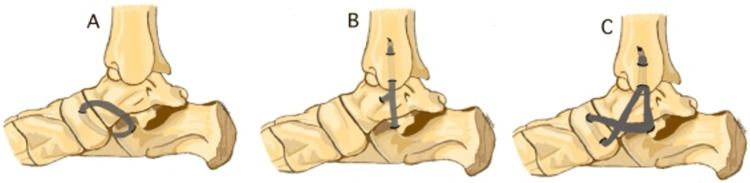
Allograft reconstruction configurations Schematic diagram of allograft reconstruction configurations used by MacDonald et al. [15] with (A) spring only, (B) deltoid only, and (C) combined tibiocalcaneonavicular ligament. Reprinted with permission from SAGE Publications: MacDonald A, Ciufo D, Vess E, et al.: Peritalar Kinematics With Combined Deltoid-Spring Ligament Reconstruction in Simulated Advanced Adult Acquired Flatfoot Deformity. Foot Ankle Int. 2020;41(9):1149-57 [15]

Twelve patients (14 feet) underwent osseous and tibiocalcaneonavicular (TCNL) reconstruction for the treatment of stage IV AAFD or stage IIB with large spring ligament tears [14]. TCNL reconstruction used semitendinosus or peroneus longus tendon allograft. The technique (Figure 2) involves passing the graft through the anterolateral tibia to the medial malleolus before attaching it to the calcaneus and navicular bones. Six feet used the schematic in Figure 3A, while eight feet used the approach in Figure 3B. The Foot and Ankle Ability Measure (FAAM), SF-36, and Patient-Reported Outcomes Measurement Information System (PROMIS) scores were used as measures of success, along with abduction and sagittal arch weight-bearing radiographs. FAAM score increased from a mean of 69.3 pre-operatively to 90.1 post-operation. SF-36 physical function subscale averaged 39.4 pre-operation, with a score of 87.8 post-operation. The pain subscale of SF-36 was 44.6 pre-operation and 93.1 post-operation. PROMIS physical function scores averaged 38.2 pre-operation and 46.8 post-operation. PROMIS pain interference scores averaged 62.6 pre-operation and 50.1 post-operation. Radiographic results found a pre-operative talo-first metatarsal angle of 24.7° with post-operation angle of 11.8°​​​​​​​. The talonavicular coverage angle was 47.4°​​​​​​​ pre-operation and 23.1°​​​​​​​ post-operation. Meary's angle averaged 29.7°​​​​​​​ pre-operatively and 12.5°​​​​​​​ post-operatively. The calcaneal pitch angle averaged 11.7°​​​​​​​ pre-operation and 16.9°​​​​​​​^ ^post-operation.

​​​​​​​

A 10-specimen cadaveric study conducted by MacDonald et al. [15] also examined the deltoid + spring ligament reconstruction treatment method. Mild, moderate, and severe flatfoot models were created by sequential sectioning of the medial capsuloligamentous complex, followed by cyclic axial loading. Treatments then consisted of spring ligament only (Figure 3A), deltoid ligament only (Figure 3B), or combined deltoid-spring ligament (TCNL) (Figure 3C). Each specimen underwent kinematic testing of all three techniques in a random order. The relative kinematic changes were then compared using a two-way analysis of variance (ANOVA). Although all medial ligament reconstruction methods were able to correct the forefoot abduction, the TCNL reconstruction was the only procedure able to correct both the subtalar and tibiotalar valgus deformity. Pre-operatively, the subtalar valgus angle averaged 5.1° in the moderate flatfoot models and 5.8°​​​​​​​ in the severe models. The subtalar valgus angles following isolated spring, isolated deltoid, and TCNL averaged 1.9°​​​​​​​ + 5.2°​​​​​​​, 3.7°​​​​​​​ + 2.8°​​​​​​​, and 0.9°​​​​​​​ + 5.7°​​​​​​​, respectively. The tibiotalar valgus angle averaged 5.1°​​​​​​​ pre-operatively in the severe models. After isolated spring, isolated deltoid, and TCNL reconstruction, the tibiotalar valgus angle averaged 2.0°​​​​​​​ + 3.5°​​​​​​​, 1.3°​​​​​​​ + 2.7°​​​​​​​, and -0.3°​​​​​​​ + 4.4°​​​​​​​, respectively.

Discussion

Although many of the included studies deemed their treatments to be a "success," this is often subjective with not a single study returning the ankle to its normal anatomical parameters except the cadaveric study by MacDonald et al. [15] with TCNL reconstruction. Deland et al. [11] considered a valgus tilt of <5° post-operatively to be a success, while no patients achieved 0° of valgus tilt. Ellis et al. [10] stated, "It is not known what threshold of talar tilt leads to satisfactory outcomes" and that they "… observed that patients that achieve and maintain <5° of tilt generally have good outcomes." Once again, no patient was able to achieve 0° of valgus tilt post-operatively, with both studies using the peroneus longus autograft in deltoid ligament reconstruction. While these two studies are used as examples, a number of other authors posit that <5° of valgus tilt is acceptable [9,12,13,15]. However, other studies have demonstrated that mild-moderate valgus malalignment at the tibiotalar joint can lead to significant ankle arthritis and/or other secondary long-term sequelae [17-20].

It is clear that some guidelines or standards are necessary for establishing a successful treatment designation. As it is well established that any imbalance in tibiotalar tilt produces a significant risk for progressive arthritis and ligamentous failure, the common goal should be to return the ankle to its normal anatomical position [17,21,22]. It has been observed in 98% of ankles that it is normal to have <2° of tilt, with >2° indicating a high probability of significant injury/reinjury to the deltoid ligament [23]. Cox and Hewes [24] even stated that "a normal ankle in a healthy young adult with no history of trauma has a small probability of having any talar tilt," making 0° of tilt the anatomical normal. To date, there appear no treatment options for chronic deltoid insufficiency in stage IV AAFD to correct valgus tilt to an anatomical standard.

Another cadaveric study by Song et al. [25] used triple arthrodesis alone in treating AAFD. While the study did not fully meet the inclusion criteria for stage IV AAFD, the findings are still relevant. The goal of this study was to determine whether triple arthrodesis alone leads to an increased strain on the deltoid complex. Following the creation of the flatfoot models, each specimen was then loaded in three gait cycle positions: heel strike, midstance, and heel rise. Components of ground reaction force and the respective tendon forces consistent with those positionings were then applied. The strain on the tibiocalcaneal fibers of the superficial deltoid ligament complex was measured at each gait position. The results of this study found a significant increase in the strain of the deltoid ligament following triple arthrodesis, most notably at the heel rise positioning. This is a significant finding when considering stage IV treatment options involving triple arthrodesis such as triple with deltoid reconstruction [7].

Another study to reference when considering triple arthrodesis in the treatment of AAFD is a cadaveric study carried out on six fresh-frozen cadavers by Resnick et al. [26]. Flatfoot deformities and deltoid insufficiency were generated in each specimen and tested in the neutral position with axial loads of 25, 50, and 75 kg. A rosette strain gauge was mounted directly onto the medial malleolus and was used to determine the magnitude and direction of the principal strain with forces on the deltoid ligament. Deltoid forces were reported as a percentage of the control. When triple arthrodesis was combined with a lateral displacement calcaneal osteotomy, forces generated in the deltoid ligament were 76% less than those measured with intact tibialis posterior tendon. Triple arthrodesis combined with medial displacement calcaneal osteotomy produced deltoid ligament forces 56% less than those exhibited following lateral displacement calcaneal osteotomy. There was no mention of post-procedural ankle alignment. Although a flatfoot with deltoid failure remains a complex problem to treat, it is clear that there also needs to be a standardization of outcome measures given the variable treatment methods available. In this study alone, we found over 25 different subjective and objective measures used to determine "treatment success." No two studies used the exact same measures, making it nearly impossible to directly compare treatment options. There needs to be a consistent way to assess outcomes when it comes to deltoid reconstruction in stage IV AAFD. 

## Conclusions

This literature review provided great insight into the status of scientific consensus on the topic of how to treat a deltoid insufficient stage IV AAFD. Unfortunately, we identified 12 studies of various types that all treated stage IV AAFD differently. Moreover, no two studies used the same set of subjective or objective/radiographic measures. The nine studies that met the inclusion criteria utilized 25 different outcome measures. Not only was there little consensus on what to measure, but there were also vastly different lengths of post-operative follow-up time for data collection. This literature review illuminates the necessity for additional research on this topic and how essential it will be to develop standard outcome measures. Without agreement on what constitutes a successful treatment, the question of efficacy seems moot. With this in mind, we encourage readers to consider that when treating stage IV AAFD, the goal should always be to return the ankle to its normal anatomical position. It is clear that this is not always possible, but that should not change the goal of recreating neutral ankle alignment.
